# Simple, fast and affordable triaging pathway for COVID-19

**DOI:** 10.1136/postgradmedj-2020-138029

**Published:** 2020-05-21

**Authors:** Elizabeth Jane Eggleton

**Affiliations:** Cardiology Department, Addenbrooke’s Hospital, Cambridge University Hospitals, Cambridge, UK; Medical School, The University of Newcastle, Newcastle upon Tyne, UK

**Keywords:** health economics, infectious diseases, molecular diagnostics, public health, chest imaging, pathology

## Abstract

Coronavirus disease 2019 has caused a global pandemic. The majority of patients will experience mild disease, but others will develop a severe respiratory infection that requires hospitalisation. This is causing a significant strain on health services. Patients are presenting at emergency departments with symptoms of dyspnoea, dry cough and fever with varying severity. The appropriate triaging of patients will assist in preventing health services becoming overwhelmed during the pandemic. This is assisted through clinical assessment and various imaging and laboratory investigations, including chest X-ray, blood analysis and identification of viral infection with SARS-CoV-2. Here, a succinct triaging pathway that aims to be fast, reliable and affordable is presented. The hope is that such a pathway will assist health services in appropriately combating the pandemic.

## Introduction

In December 2019, a novel type of viral pneumonia emerged in Wuhan, China, which was later named Coronavirus disease (COVID-19).^1–3^ COVID-19 is caused by the virus severe acute respiratory syndrome corona 2 virus 2 (SARS-CoV-2), which can lead to acute respiratory disease. This is associated with highly non-specific symptoms of fever, dry cough and dyspnoea.^4–7^ In around one in five people, this leads to severe illness and requires admission to hospital for supportive care.^8–10^ This clinical decision of whether a patient requires admission or discharge is made difficult due to the highly idiosyncratic nature of the virus. Nonetheless, this decision is increasingly important due to the demand that the pandemic poses to health services and the need to optimise the utilisation of available health resources. The aim herein is to assist frontline clinical staff in the triaging of patients through a fast, reliable and affordable pathway.

## Clinical assessment

**Figure 1:**
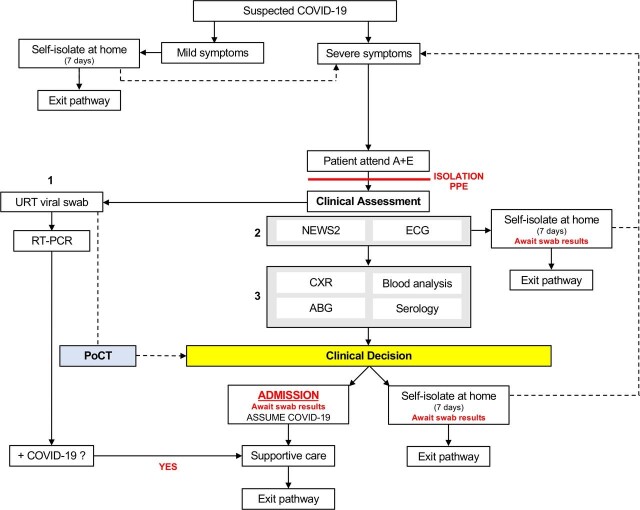
Pathway to triage patients with COVID-19 from initial presentation. Decision points are discussed in more detail in the main text. (1) Viral swab collection; (2) initial assessment; (3) further investigations. ABG, arterial blood gas; A+E, Accident and Emergency; CXR, chest X-ray; NEWS2, National Early Warning Score 2; PoCT, point-of-care test; PPE, personal protective equipment; RT-PCR, Reverse-Transcriptase PCR; URT, upper respiratory tract.

Current government advice recommends that patients displaying mild symptoms of COVID-19 should self-isolate at home for 7 days unless these symptoms become severe (figure 1). Mild symptoms typically present as an upper respiratory tract infection that causes common non-specific symptoms of fever, dry cough and dyspnoea. However, these symptoms can become more severe due to development of viral pneumonia and require medical attention. Patients are presenting at emergency departments with a spectrum of symptoms that are compatible with a severe respiratory infection. This requires patients to be isolated on arrival by healthcare professionals wearing appropriate personal protective equipment (PPE). Initially, a clinical history should be taken by a clinician in order to determine the patient’s symptoms and the presence of any underlying health conditions. Following this, all patients who present with symptoms indicative of COVID-19 should undergo a viral swab (figure 1, step 1).

### Upper respiratory tract swab: SARS-CoV-2 identification

The standard diagnosis of active viral infection is achieved through reverse-transcriptase PCR (RT-PCR). This is the gold-standard test in identifying an active SARS-CoV-2 infection.^4 11^ It is achieved through identifying presence of the SARS-CoV-2 virus within respiratory secretions and is initially performed from an upper respiratory tract sample, with nasopharyngeal having preference to oropharyngeal.^12 13^ RT-PCR is reported to be highly specific, but the sensitivity varies from 59% to 71%.^14 15^ This could be due to the optimum time of sample collection during the viral infection not currently being known.^16^ Furthermore, the infection has been shown to target the lower airways and a positive test may be acquired from a lower respiratory tract sample. This may not always be appropriate due to the aerosol-generating nature of obtaining this sample.

The time required to complete this laboratory investigation has been reported to be up to 72 hours, which means that it cannot currently complement the clinical decision whether to admit or discharge patients. Nonetheless, the result from this test is important in the management of the patient and for public health considerations (figure 1). The ability to use a point-of-care test (PoCT) that is a quick and reliable method to test patient samples would contribute to the clinical decision in the triaging process. Recently, success has been reported in developing a technique of reverse transcription loop-mediated isothermal amplification (RT-LAMP).^5 17–20^ This test is a one-step nucleic acid amplification technique, which can be performed at ambient or low temperature and can speed the process to 10 min.^4 17^ RT-LAMP is a highly sensitive and specific test for COVID-19 and has shown ability to become a PoCT (figure 1).^5 21^ The implementation of RT-LAMP-based tests for virus detection is more appropriate as a frontline diagnostic test due to the current high demand of tests, reduced time to result and low cost of the test.^5 17^ There is also the potential to incorporate a smartphone application, which was used during the Zika virus outbreak, in order to share data directly with WHO and Public Health England.^20^

However, while the identification of SARS-CoV-2 is important for the appropriate management of the patient, the result cannot currently contribute to the triaging process. Therefore, the triaging process relies on clinical assessment, laboratory and imaging investigations (figure 1). These are split into an ‘initial assessment’, which aims to identify acutely ill patients and ‘further investigations’, which further contributes to the clinical picture. Each investigation will be outlined and its contribution to the assessment of patients with suspected COVID-19 will be discussed.

### Initial assessment

#### National Early Warning Score 2

After patients have had a viral swab an initial assessment should be completed (figure 1, step 2). This is guided through the National Early Warning Score 2 (NEWS2) assessment, which allows identification of acutely ill patients through the assessment of respiration rate, oxygen saturation, temperature, systolic blood pressure, heart rate and level of consciousness.^22^ Patients may have tachycardia, hypoxia and tachypnoea associated with respiratory distress caused by COVID-19. Furthermore, the identification of hypertension has recently been identified as an independent risk factor for disease severity.^23^ A higher NEWS2 score indicates a more acutely unwell patient and this assists the decision as to whether further tests are required. A NEWS2 score more than three indicates an acutely unwell patient and this would indicate the need for further investigations (figure 1, step 3).

#### Electrocardiogram

In addition to NEWS2 assessment, the patient should have an ECG. While the ECG is not a direct diagnostic tool for COVID-19, it allows differential diagnoses to be ruled out, such as myocardial infarction. This is a quick, easy and low-cost test that should be used on all patients presenting with symptoms of dyspnoea.

After the initial assessment is performed, the clinicians make a decision whether the patient requires further investigations. At this point, if the patient is not acutely unwell, it is possible to advise to self-isolate for 7 days and await swab results. Patients are advised if their symptoms worsen they should seek medical assistance again.

### Further investigations

#### Chest X-ray

Clinicians should request a chest X-ray; a useful frontline diagnostic imaging technique that uses ionising radiation to identify changes within the lung parenchyma.^24 25^ Other countries, such as China, have used High Resolution Computed Tomography (HRCT) as the primary imaging modality for COVID-19 assessment.^26^ Various features have been identified in patients with COVID-19 from chest X-ray and HRCT (table 1).

**Table 1:** Common imaging features identified in HRCT and Chest X-ray of patients with COVID-19

Imaging features identified in patients with COVID-19	Reference
Consolidation	4 33–35
Bilateral involvement	4 24 25 33–35
Peripheral and diffuse involvement	12 25 33–35
Ground-glass opacification	4 12 25 33–35

HRCT, High Resolution Computed Tomography.

The use of chest X-ray in triaging patients is appropriate as it is a quick, accessible and cost-effective technique to assess for viral pneumonia.^27^ On chest X-ray, clinicians need to assess for consolidation and peripheral and diffuse involvement, which has also been reported to be bilateral in nature. The identification of these features indicates viral pneumonia (table 1), which is not sufficient for a diagnosis of COVID-19 as the results are highly non-specific. However, it is essential in contributing to the clinical decision.

Chest X-ray is not highly sensitive either and therefore the lack of these features does not rule out the diagnosis of COVID-19. HRCT is a more sensitive imaging modality, which is able to identify ground-glass opacification that may not be visualised on chest X-ray.

It has been suggested that HRCT can provide benefit to the diagnosis of COVID-19 when swab results are negative.^15^ A retrospective study of 1014 cases in China suggested that RT-PCR is less sensitive than HRCT in diagnosing COVID-19 and suggested that HRCT be considered as a primary diagnostic tool.^15^ This is supported by further research that advised that HRCT is more sensitive than RT-PCR (98% vs 71%, respectively, p<0.001) and therefore it is suggested that it could be used to screen patients with clinical features of COVID-19 during triage.^14^

However, the use of HRCT exposes the patient to 100 times more radiation than chest X-ray and is a more expensive test.^27^ In the current situation with increasing numbers of patients presenting at hospital, its increased cost means that it is not necessarily an appropriate frontline diagnostic technique. In addition, while having a higher sensitivity than chest X-ray,^14 15^ it also has low specificity.^14^ Furthermore, infection control of using this equipment with patients with suspected COVID-19 and the associated risk of cross-infection must be considered.^28^ Therefore, its limitations do not outweigh the benefits to be used as a triaging technique. It instead may be used later in the treatment pathway if it is likely to impact the clinical management of the patient.

#### Blood analysis

Blood analysis is a central component to patient triaging during COVID-19. The specific tests that should be ordered are outlined elsewhere.^26^ The analysis of blood results in patients diagnosed with COVID-19 has identified some common features that are altered (table 2).

**Table 2:** Common features present in blood analysis of patients with COVID-19

Blood component	Indication in COVID-19	Reference
CRP	Elevated	4 23 25 26
Lymphocytes	Decreased	4 10 12 23 36 37
Leukocytes	Normal or decreased	7 10 37
Neutrophils	Elevated	7
D-dimer	Elevated	7 37 29
Serum troponin	Elevated	7
Liver function tests	Elevated	10 26

CRP, C reactive protein.

The most significant hallmarks noted are raised C reactive protein (CRP) and lymphopenia, which are consistent with the inflammatory response produced by a virus. Various studies have reported a raised CRP and lymphopenia in patients with COVID-19, and this is associated with more severe illness and patients requiring longer hospital admissions.^23^ Therefore, assessing CRP and lymphocyte count is a central component to triaging patients.^26^ In line with the inflammatory response, elevated neutrophils have been reported (table 2). Furthermore, identification of elevated D-dimer at admission is associated with increased disease severity and mortality.^29^

The interpretation of blood results is an important component of the triaging pathway and identification of key features, raised CRP and lymphopenia, allows for the indication of patients with likely COVID-19 infection. Retrospective studies should be performed to identify whether there are indicators of disease severity on arrival at the emergency department, which will further assist the triaging process in future.

#### Arterial blood gas

The measurement of arterial oxygenation is achieved through performing arterial blood gas (ABG) testing. This can assess hypoxia, hypercarbia and acidosis, which contributes to the clinical picture and identification of acute respiratory distress syndrome (ARDS). ARDS commonly develops in the setting of pneumonia and is a complication reported in COVID-19.^8^ The use of ABG is a quick tool that can detect whether the patient requires oxygen therapy.^7 30^

#### Serological testing

Serological assays to detect infection of COVID-19 are currently in development.^31^ This is achieved through identifying the acute profile of antibodies produced by the bodies humoral and cellular immunity against SARS-CoV-2.^6^ The serological assay, ELISA, is designed to detect the presence of IgM and IgG antibodies and with detection.^13 32^ Currently, serological testing is being introduced into testing in the acute emergency department setting. However, the median time taken for seroconversion of IgG and IgM has been shown to be up to 13 days after symptom onset, which may limit its applicability as a frontline diagnostic test.^32^ Serological testing also has the ability to be a useful tool in retrospectively confirming patient exposure to the SARS-CoV-2 virus.

## Clinical decision

The results from all of these tests will form a clinical picture of the patient that has attended the emergency department. The clinical decision is made with consideration of all of the results from these tests. While the result of the viral swab for SARS-CoV-2 does not assist the clinical decision-making, the development of a PoCT could assist this. This would also relieve patient anxiety in relation to waiting for test results, which is important when considering the holistic care of the patient.

The variety of tests presented here are cost-effective, informative and relatively quick tests in order to contribute to comprehensive clinical evaluation. This will assist in the decision of whether the patient requires admission or if they are able to continue to self-isolate at home awaiting swab results and return if symptoms worsen (figure 1). Admission to hospital allows for supportive care to be provided to the patient until they either recover or end-of-life care is required.

## Conclusion

This pathway is intended as a simple, rapid and cost-effective workflow tool that can be used as an overview of the triaging process of patients presenting with symptoms indicating COVID-19. The implementation of such a strategy will aim to reduce the strain on the healthcare services during the current pandemic. However, as always, it is paramount to consider individual patient circumstances when making clinical decisions and this pathway should aid, not replace, clinician decision-making.

**List of learning points**

- The importance of appropriate triaging during the pandemic with limited health resources
- How the process of triaging patients with symptoms indicative of COVID-19 can be achieved
- The use of various laboratory and imaging techniques in the diagnosis of COVID-19
- The common identified features of SARS-CoV-2 infection in blood analysis and in the lungs using HRCT, chest X-ray
